# The Molecular Mechanisms of Cold Tolerance in *Brassica napus*

**DOI:** 10.3390/ijms27219905

**Published:** 2026-11-05

**Authors:** Weiqi Luo, Yan Zhou, Jinyu Shen, Lei Xu, Xuezhu Du, Feng Sheng, Rudan Geng, Guangqin Cai

**Affiliations:** 1 Key Laboratory of Biology and Genetic Improvement of Oil Crops, Ministry of Agriculture and Rural Affairs, Oil Crops Research Institute, Chinese Academy of Agricultural Sciences, Wuhan 430062, China; 17371650637@163.com (W.L.); z2022609243@163.com (Y.Z.); 15827072101@163.com (J.S.); 15202733352@163.com (L.X.); 2 School of Life Science, Hubei University, Wuhan 430062, China; duxuezhusk@163.com (X.D.); shengfsk@163.com (F.S.); 3 Hubei Hongshan Laboratory, Wuhan 430070, China

**Keywords:** *Brassica napus* L., low-temperature stress, physiological and morphological response, molecular mechanism, elite germplasm

## Abstract

*Brassica napus* is a crucial oilseed crop in China, meeting approximately 55% of the country’s demand for vegetable oils. However, throughout its growth cycle, it frequently encounters low-temperature stress. As a key environmental factor constraining the stability of *Brassica napus* L. yield and quality, cold damage makes research into the cold tolerance mechanisms of *Brassica napus* L. highly significant for the breeding of cold-tolerant varieties. This paper focuses on the issue of low-temperature stress in *Brassica napus* L., synthesizing recent relevant research findings. It first clarifies the phenotypic effects of low-temperature stress on *Brassica napus* L. at different growth stages. Concurrently, low temperatures disrupt the integrity of the *Brassica napus* cell membrane and inhibit photosynthesis, in response, plants can mitigate oxidative damage by activating antioxidant enzyme systems, such as superoxide dismutase (SOD) and catalase (CAT). Based on the above, the current research progress in mining cold-tolerant germplasm resources in China and identifying cold tolerance genes in *Brassica napus* L. is further summarized as follows. This paper provides a theoretical reference for an in-depth analysis of the cold tolerance mechanisms in *Brassica napus* L. and for accelerating the breeding of cold-tolerant varieties. Future efforts should focus on the functional validation of key genes within cold tolerance pathways and the application of multi-gene pyramiding breeding technologies to break through the bottleneck in controlling cold damage in *Brassica napus* L.

## 1. Introduction

Temperature plays a crucial role in the growth and development of plants. Among various abiotic stress, low-temperature stress affects most plants globally, impacting multiple aspects such as their growth, development, morphology, and yield [1], leading to severe consequences such as plant damage or even death [2]. The damage caused by low temperatures to plants is mainly categorized into two types: chilling injury (0–15 °C) and freezing injury (<0 °C) [3,4]. Chilling injury impairs plant growth and development in several ways. It reduces the synthesis of photosynthetic assimilates, delays growth, inhibits nutrient uptake by the roots, and causes poor development of reproductive organs. In severe cases, it can lead to plant death [5,6]. Freezing injury, a type of agricultural meteorological disaster, occurs when temperatures fall below 0 °C, leading to the formation of ice within crops and consequently affecting their health [7], damage intensifies as the freezing period lengthens and may become irreversible in severe [8]. To cope with freezing stress, plants in temperate climates have evolved complex cold acclimation mechanisms. When exposed to non-freezing low temperatures, this mechanism enhances plant freezing tolerance and commonly occurs in crops such as rapeseed, winter wheat, and barley [1]. In China, freezing injury poses a serious threat to agriculture, particularly in the northwest, north, east, and central-south regions [9].

Chilling stress refers to exposure to low but non-freezing temperatures, typically above 0 °C, and its primary effect is to disturb membrane physical state and metabolism rather than to cause mechanical damage. In *B. napus*, cold-induced membrane rigidification is itself sufficient to activate cold-responsive genes, as shown by the suppression of *BN115* activation with a fluidizer and its mimicry at 25 °C with a rigidifier [10]. Chilling also depresses photosynthetic electron transport and carbon assimilation, perturbs enzyme kinetics and induces oxidative stress. These effects are largely reversible: growth and metabolism recover once temperatures return to the permissive range, and the injury reflects a shift away from an optimum rather than destruction of cellular structure.

Freezing stress occurs when temperatures fall below 0 °C and ice forms, and injury arises chiefly from the mechanical and osmotic consequences of ice nucleation. Extracellular ice lowers water potential and dehydrates the cell, so the plasma membrane must shed a large proportion of its surface area and re-expand on thawing without rupturing. Acclimated cells tolerate a greater area increase before lysis and store excess membrane in retrievable extrusions, whereas unacclimated cells internalize it irreversibly and lyse [8]. Intracellular ice formation is generally lethal. Freezing injury is therefore irreversible once the lytic threshold is exceeded, which distinguishes it mechanistically from chilling injury.

*Brassica napus*, a member of the Brassicaceae family within the genus *Brassica*, ranks as the oilseed crop with the largest cultivation area in China and serves as a primary source of edible oil, holds significant influence in both global trade and agricultural production [11]. Currently, China’s self-sufficiency rate for edible oil is less than 36.62%, and it remains heavily reliant on imports [12]. Therefore, expanding rapeseed production is key to raising China’s self-sufficiency in edible oil. In southern China, approximately 17.3 million ha of winter-fallow land remain available for more than 100 days, and 14.0 million ha for more than 120 days. Of this area, an estimated 7.0 million ha are suitable for a rice–rice–rapeseed rotation [13], and within the 6.7 million ha of winter-fallow land in the Yangtze River Basin, 4.3 million ha are suitable for rapeseed cultivation [14].

Temperature, as a key environmental factor determining plant growth, development, and geographical distribution [15,16]. The optimal temperature range for rapeseed growth is between 9 and 21 °C, but rapeseed suffers freezing injury when overwintering temperatures drop below approximately −3 °C; when temperatures range from −5 °C to −3 °C and persist for more than three days, the leaves of rapeseed also exhibit symptoms of freezing damage; when temperatures fall below −7 °C, rapeseed will sustain severe, irreversible damage [17]. Additionally, strong winds exacerbate the symptoms of freezing injury in rapeseed. In the cold and arid regions of northern China, meteorological and environmental factors have led to a scarcity of rapeseed cultivation area. Low temperature is the primary limiting factor for cultivation, compounded by a lack of cold-tolerant rapeseed varieties. This situation significantly impacts the local production volume and quality of edible oil in northern China [18].

With the rapid advancement of modern molecular biology and genetic engineering technologies, research on plant cold tolerance mechanisms has become a prominent focus in the biological sciences and has achieved significant breakthroughs. Notably, considerable progress has been made in plants such as *Oryza sativa* and *Arabidopsis thaliana*. With continuous exploration of genes, an increasing number of gene functions are being elucidated. In this process, certain genes have been subjected to high-frequency research, confirming their core roles in cold tolerance regulation. These genes generally contribute to the molecular network through which plants respond to cold stress by regulating membrane stability, activating cold signaling pathways, modulating osmolyte synthesis, and scavenging reactive oxygen species (ROS) [19]. The perception of low temperature initiates the cold stress response, a step that links stimulus perception to the activation of downstream resistance mechanisms [9]. On chromosome 4 of the *Arabidopsis thaliana* genome, three members of the C-Repeat Binding Factor (CBF) gene family, namely *CBF1*–*CBF3*, are arranged in tandem within an 8.7 kb region [20,21]; Stockinger et al. [22] first cloned *CBF1* (*AtCBF1*) from *Arabidopsis thaliana*. Subsequently, *AtCBF2* and *AtCBF3* were successively identified. Following this, homologous genes have been cloned in various plants such as rice, wheat, maize, and rapeseed, with their sequences being highly conserved (all containing the DNA-binding domain characteristic of AP2/ERF transcription factors) [23,24]. Under cold stress, CBF genes are rapidly induced and expressed, the transcription factors can specifically bind to the CRT/DRE cis-elements in the promoters of downstream target genes, activating the expression of hundreds of cold-tolerance-related genes, such as *Cold-Regulated* (COR), *Dehydration-Responsive* (RD), *Cold-Inducible* (KIN), etc., thereby enhancing plant cold tolerance through multiple pathways [25,26]. Particularly, the *COR* genes are core target genes of the CBF pathway, the cold-regulated proteins they encode serve as direct effector molecules for plants in response to low temperature [27]. Thomashow et al. [3] isolated four COR cold-tolerance genes from *Arabidopsis thaliana*, including *COR15a*, *COR616*, *COR78*, and *COR47* [22]. Among them, COR15a is a small hydrophilic protein primarily localized to the chloroplast stroma and thylakoid membrane. Research has found that under low temperatures, the expression of *COR15a* is significantly upregulated, and its main function is to enhance the low-temperature stability of thylakoid membranes [28]. COR15a can interact with membrane phospholipids, reducing the membrane lipid phase transition induced by low temperatures, thereby maintaining membrane integrity and permeability [29]. *The Inducer Of CBF Expression 1* (*ICE1*) gene, serving as the “upstream regulator” of the CBF pathway, is a key upstream transcription factor that regulates *CBF* gene expression and is an indispensable gene in plant cold tolerance research [30], *ICE1* is a bHLH-type transcription factor localized in the nucleus and exists primarily in an inactive form under normal temperatures [31]; under cold stress, *ICE1* is activated through post-translational modifications such as phosphorylation and ubiquitination, subsequently binding to the promoter regions of the *CBF1* and *CBF3* genes to promote their transcription and expression [32,33]. The function of *ICE1* is highly conserved in plants, for example, overexpression of *VcICE1* in blueberry or *MdICE1* in apple can significantly enhance the cold tolerance of transgenic plants, with both achieving this function by regulating downstream homologous *CBF* genes [34,35]. Simultaneously, there exist transcriptional repressors that directly inhibit cold-induced CBF activation, such as *Myeloblastosis 15* (*MYB15*). *MYB15* is a member of the MYB transcription factor family and can regulate CBF expression under low-temperature conditions [25]. Current research also indicates that *Mitogen-Activated Protein Kinase 6* (*MPK6*) can interact with and phosphorylate *MYB15*, thereby reducing its ability to bind to CBF promoters [24], and then alleviating the inhibitory effect of this transcription factor on *CBF* gene expression. These findings indicate that during cold acclimation, negative transcription factors also play an important role in maintaining the homeostasis of the transcriptional network regulated by *CBF*. Focusing on the *CBF* cold response pathway across multiple species beyond *Arabidopsis thaliana*, it has been discovered that both cold-acclimating plants (such as rapeseed and wheat) and non-acclimating cold-sensitive plants (such as tomato) possess CBF-like genes, the proteins they encode contain conserved functional motifs [20]. Under low temperatures, CBF transcripts rapidly accumulate, overexpression of *Arabidopsis thaliana CBF* genes in rapeseed can induce the expression of downstream cold-regulated genes and enhance cold tolerance. Although tomato possesses CBF-like genes, their transcript levels cannot be sustained at high levels under cold stress, which may explain its inability to cold acclimate. These discoveries collectively indicate that the components of the CBF cold response pathway are highly conserved in angiosperms and are not exclusive to cold-acclimating plants, providing a theoretical basis for improving cold tolerance in crops [36].

## 2. Impacts of Low-Temperature Stress on *Brassica napus*

The impact of cold stress on the growth and development of *Brassica napus* is highly significant and manifests throughout its entire physiological process. The growth objectives and sensitivity to low temperature vary markedly across different growth stages of rapeseed, and consequently, the mechanisms of cold stress effects and the severity of damage also differ.

The germination capacity, germination speed, and seedling establishment potential of rapeseed seeds under low temperatures serve as the core criteria for screening cold-tolerant germplasm resources during the germination stage [37]. By tracking the seed germination process and final status, the inhibitory effect of low temperature on seed germination can be directly assessed. Additionally, observing the final seedling establishment rate reflects the extent of seed damage caused by low temperature [6]. Studies subjecting different rapeseed varieties to cold treatments have found that as temperature decreases, germination and emergence rates of all varieties slow down, and the seedling vigor index (which was calculated as germination percentage multiplied by radicle length) also declines [38].

Water and temperature are core environmental factors regulating the seed germination process, acting synergistically on key physiological stages: seeds first complete water absorption through imbibition, softening the seed coat and restoring metabolic activity in the embryonic cells, laying the groundwork for subsequent metabolism. The temperature conditions during the germination stage directly determine the efficiency of enzymatic reactions within the seed [39]—when temperatures are too low, the activity of hydrolytic enzymes such as amylase and lipase is significantly inhibited, leading to a decreased breakdown rate of energy storage substances like starches and fats. These results in insufficient nutrients and energy for embryo growth. Concurrently, low temperatures disrupt membrane fluidity, hindering material transport and gas exchange (e.g., oxygen uptake, carbon dioxide release) across cell membranes [40]. This further inhibits morphological processes such as radicle protrusion through the seed coat and hypocotyl elongation, ultimately causing delayed germination, reduced germination rates, or even germination arrest [41].

The survival rate of rapeseed at the seedling stage is the most direct indicator for measuring its cold tolerance threshold. It is typically determined through experiments involving recovery growth under normal conditions after cold treatment. Specifically, rapeseed plants at the 3–4 leaf stage are placed in an artificial climate chamber, subjected to a set period of cold treatment, and then transferred back to normal growth conditions for recovery. Survival is scored based on the ability to produce new leaves and resume normal growth. Research shows that survival rates at the seedling stage vary significantly among varieties.

Low temperature inhibits photosynthesis and biomass accumulation in rapeseed, and reduces plant height, root and shoot fresh and dry weights. Among these, biomass accumulation capacity also serves as an important indicator reflecting varietal cold tolerance [42]. Under cold stress, cold-tolerant materials enhance their water and nutrient uptake capacity by prioritizing the maintenance of root growth, resulting in a higher root-to-shoot ratio. During treatment, cold-tolerant varieties exhibit a slower decline in growth rate and can rapidly achieve compensatory growth after recovery, whereas sensitive varieties are prone to a growth arrest response [43].

Leaves are the primary photosynthetic organs in rapeseed and the foremost site of low-temperature damage, changes in their morphology and structure can intuitively reflect the plant’s cold tolerance [44]. Under cold stress, chlorophyll degradation leads to leaf yellowing, and in severe cases to anthocyanin accumulation. Low temperatures also cause leaf curling, which reduces leaf area and thereby limits transpirational water loss. Cold-tolerant genotypes generally show milder curling that is reversible after transfer to normal growth conditions, whereas sensitive genotypes show severe curling accompanied by irreversible damage [45].

The flowering stage is a critical period for rapeseed yield formation, low temperatures (2–5 °C), while not causing plant death, can severely affect floral organ development and the pollination process. The morphological phenotypes of floral organs are directly linked to seed set rates and yield [46]. Under low temperatures, the primary observation focuses on chilling injury symptoms in the plant’s upper parts, including the bolting stem leaves, flower buds, petals, main inflorescence, branch inflorescences, pedicels, young siliques, and mature siliques [47].

In contrast to vegetative stages, cold sensitivity during seed filling has rarely been examined directly in rapeseed. Available evidence indicates that low night-time temperature during seed filling alters oil quality by shifting the fatty acid profile towards elongation rather than enhancing desaturation, lowering oleic acid while raising erucic and linolenic acids [48]. Seed-filling temperature therefore reshapes oil quality through competition between desaturation and elongation. However, oil content and hardiness traits were not assessed, and the treatment was a mild night-time cooling rather than a cold stress, so the consequences for both oil quality and seed-lot performance remain to be quantified.

Overwintering survival reflects performance across the whole overwintering season and is strongly region-dependent. In northwest China, at altitudes of 1477–2300 m where absolute minimum temperatures range from −22.7 to −31.6 °C, *B. napus* accessions largely fail to overwinter, while *B. rapa* accessions exhibit high survival rates. This delineates a species-level boundary for winter rapeseed cultivation. Within *B. napus*, field overwintering rate is closely associated with LT_50_ determined in controlled freezing tests, supporting the use of laboratory-assessed freezing tolerance as a proxy for winter survival [49].

Deacclimation, the loss of acquired freezing tolerance during warm spells, has been examined in only a few studies of winter rapeseed, although it is the stage most directly coupled to winter climate variability. A warming period reduced freezing tolerance in all tested lines without returning them to the unacclimated level, and tolerance was restored after re-exposure to low temperature, indicating that deacclimation is incomplete and reversible [50]. This partial reversibility is evident at the physiological level: after a week of warming, leaf ABA declined from its acclimated maximum but remained above non-acclimated levels [51], and proline in leaves and root crowns also remained well above non-acclimated values. The most pronounced change concerns water transport. Aquaporin genes were strongly repressed during acclimation and re-activated during deacclimation, with root aquaporin transcripts rising above non-acclimated levels; these transcripts correlated negatively with regrowth after freezing, whereas proline and cell-sap ABA correlated positively. Acclimated plants therefore operate in a high-protection, low-water-transport state, and deacclimation shifts them to a low-protection, high-water-transport state that favors growth resumption but leaves them vulnerable to subsequent frost [52]. In *Arabidopsis*, metabolomic profiling further showed that deacclimation-specific metabolites represent only a small fraction of the responsive metabolome, confirming that deacclimation is not simply the reverse of acclimation [16].

## 3. Physiological Indicators

Physiological functional phenotypes reveal the intrinsic mechanisms through which rapeseed adapts to low temperatures via metabolic regulation. These include core indicators for analyzing cold tolerance mechanisms, such as cell membrane protection, maintenance of photosynthesis, and antioxidant defense.

The cell membrane is the primary target of low-temperature damage, and its stability directly determines cell survival, key indicators include relative electrolyte leakage (EL) and the content of osmolytes (such as soluble sugars and proline). Low temperature increases cell membrane permeability, leading to the leakage of electrolytes such as K^+^ and Ca^2+^, resulting in an increase in relative electrolyte leakage [53]. Soluble sugars and proline are primary osmolytes, they function by lowering the cellular freezing point and stabilizing protein structures [54]. Under low temperatures, rapeseed actively accumulates these osmolytes to protect the integrity of the cell membrane [55].

Low temperatures inhibit the activity of photosynthetic enzymes and damage photosynthetic structures, leading to a decline in photosynthesis. Photosynthetic function indicators reflect the energy accumulation capacity of rapeseed under low-temperature conditions. Core indicators include chlorophyll content, chlorophyll fluorescence parameters, and net photosynthetic rate (P_n_) [56]. Under low temperatures, the rate of chlorophyll degradation serves as a crucial indicator of rapeseed variety cold tolerance [57]. Furthermore, the decrease in net photosynthetic rate results from either insufficient carbon dioxide (CO_2_) supply due to reduced stomatal conductance (G_s_), or from a decline in photosynthetic enzyme activity [58]. Under cold stress, key parameters in plant leaves decline, such as the maximum photochemical efficiency of PS-II (*F_v_*/*F_m_*), the efficiency of excitation energy transfer to open PS-II reaction centers (*F_v_*_′_/*F_m_*_′_), and the photochemical quenching coefficient (qp), while Photosystem II PSII simultaneously experiences photoinhibition [59,60].

Low temperature induces the accumulation of ROS, such as superoxide anions and hydrogen peroxide, excessive ROS damages cellular structures [61]. The antioxidant enzyme system (including SOD, POD, CAT) is crucial for scavenging ROS, and its activity directly reflects the antioxidant capacity of rapeseed [62,63]. Superoxide dismutase (SOD) serves as the “first line of defense” in the redox system, responsible for scavenging superoxide anions. Peroxidase (POD) and catalase (CAT) work synergistically to scavenge hydrogen peroxide [64], indicating that strong antioxidant capacity is an important support for membrane protection.

Two caveats should be borne in mind when interpreting these antioxidant data. First, ROS accumulation and the accompanying induction of SOD, POD and CAT are not specific to cold: the same response is elicited by drought, salinity, heat and heavy-metal stress, and these enzyme activities are therefore widely used as markers of a general abiotic-stress response rather than of cold tolerance per se. Second, the relationship between antioxidant capacity and cold tolerance is contingent on timing, tissue and stress severity. Activities measured at a single time point do not necessarily reflect the actual capacity to scavenge ROS, and they become informative only when read together with functional endpoints such as electrolyte leakage, MDA content and LT_50_. In *B. napus*, increases in SOD and CAT activity should therefore be interpreted as evidence of an activated stress response, whose contribution to tolerance has to be established independently rather than inferred from activity alone.

Phytohormones are a class of small-molecule compounds crucial for regulating plant growth and development. Even at trace concentrations, they exert significant regulatory effects [65]. The fluctuation amplitude of endogenous phytohormone levels in leaves in response to temperature changes is significantly smaller in strongly cold-tolerant *Brassica napus* winter varieties/lines compared to their weakly cold-tolerant counterparts [66]. Abscisic acid (ABA), as a primary stress-responsive phytohormone, accumulates under cold stress. This accumulation subsequently reduces potassium ion content in cells, leading to stomatal closure. Gibberellin (GA), as a key class of phytohormones, can regulate plant cold tolerance by modulating the dynamic balance between plant growth/development and stress responses. Under cold stress conditions, endogenous GA levels typically decline. This low GA state can effectively enhance cold tolerance during the plant’s growth stages [67]. Auxin (IAA) not only participates in regulating multiple processes of plant growth and development but also plays a significant role in plant responses to cold stress. Under low-temperature conditions, plants can enhance their adaptability to stress by downregulating endogenous IAA levels [68,69].

Analysis of the changes in endogenous phytohormone levels in winter rapeseed genotypes differing in cold tolerance under low-temperature stress showed that leaf IAA, zeatin (ZT), and GA_3_ contents decreased, whereas ABA content increased 2- to 3-fold relative to the control [70]. Jasmonic acid (JA) is a critical upstream regulator of the CBF/DREB1 signaling pathway and positively regulates plant cold tolerance in *Arabidopsis thaliana* [26]. Research findings indicate that an appropriate concentration of exogenous methyl jasmonate (MeJA) can effectively alleviate cellular damage in rapeseed seedlings under low temperature, enhance their photosynthetic capacity, and protect cell wall integrity. Moreover, exogenous MeJA can induce the expression of cold tolerance genes and Ca^2+^ signal transduction in rapeseed seedlings under cold stress. This process, involving exogenous MeJA in mitigating low-temperature damage, can further influence and stimulate various physiological and biochemical responses in the seedlings, thereby enhancing their cold tolerance [71]. These combined actions comprehensively improve plant cold tolerance [72].

The plasma membrane is the earliest site of cold injury and a site of cold perception. In *B. napus*, cold activation of *BN115* was suppressed by a membrane fluidizer and mimicked at 25 °C by a rigidifier, showing that reduced membrane fluidity is itself sufficient to trigger the cold response [10]; rigidification is thought to gate Ca^2+^ permeable channels. Acclimation partly offsets this by increasing acyl unsaturation and raising the PE/PC ratio, yet restores only part of the lost fluidity, so homeoviscous adaptation remains incomplete [73]. The membrane must also withstand the mechanical consequences of freezing: acclimated cells accommodate a greater increase in surface area before lysis and store excess membrane in retrievable extrusions, whereas unacclimated cells internalize it irreversibly [8]. Finally, the polyunsaturated species produced during acclimation are themselves substrates for lipid peroxidation, and membrane damage is greater in cold-sensitive than in cold-tolerant rapeseed [17].

The channels that carry this Ca^2+^ influx have only recently begun to be identified. In *Arabidopsis*, the mechanosensitive channels MCA1 and MCA2 mediate part of the cold-induced rise in cytosolic Ca^2+^: the response is reduced in *mca1* and *mca2* single mutants and further reduced, though not abolished, in *mca1 mca2* double mutants, which are hypersensitive to chilling and freezing and fail to acquire full acclimation capacity [74]. They therefore operate within a redundant system that also includes *ANNEXIN1* and *ANN4* [75] and, in rice, the cyclic nucleotide-gated channel OsCNGC9 [76]. The OSCA family, defined by the hyperosmolality-gated channel OSCA1 [77], shares this mechanosensitive logic, but its genetic evidence concerns osmotic rather than cold stimuli, and a cold-specific function has not been established. In *B. napus*, the molecular identity of these channels is still unresolved: no MCA orthologue has been functionally characterised, and the 41 *BnaOSCA* genes identified at the genome level [78] have not been tested under cold, although cold activation of *BN115* requires both Ca^2+^ influx and protein kinase activity [10].

Plant cold acclimation is highly dependent on membrane lipid remodeling. Cold-activation of *BN115* was strongly inhibited by the membrane fluidizer benzyl alcohol, but mimicked at 25 °C by the membrane rigidifier dimethylsulfoxide (DMSO) [10]. The *Arabidopsis fad2* mutant, defective in oleate desaturase, exhibits membrane rigidification and activation of diacylglycerol kinase at 18 °C, whereas wild-type plants require 14 °C, indicating an elevated threshold for cold perception [1]. Membrane rigidification is proposed to activate mechanosensitive and/or ligand-gated Ca^2+^-permeable channels, triggering Ca^2+^ influx and initiating Ca^2+^-dependent signaling cascades [4]. In an EMS mutant population of *Brassica napus*, cold-tolerant lines showed a significantly higher proportion of unsaturated fatty acids than cold-sensitive lines, consistent with elevated expression of fatty acid biosynthetic genes including *FAD8*, *FAB2* and *KCS13* [79]. Current evidence in rapeseed remains largely correlative, and quantitative analyses linking fatty acid composition to LT_50_ have not, to our knowledge, been reported. In grape, a 6 bp InDel in the *VaFAD5* promoter creates a DOF binding site that underlies the haplotype dosage effect on cold tolerance, accompanied by elevated C18:1, C18:2 and the unsaturation index (IUFA) [80]. This study dissects a complete causal chain from cis-regulatory variation to desaturase expression, fatty acid unsaturation and cold tolerance in grapevine; an equivalent chain has not, to our knowledge, been established in rapeseed.

Membrane lipid remodeling. Quantitative evidence for lipid remodeling in *B. napus* comes from endoplasmic reticulum membranes of etiolated hypocotyls, where cold acclimation raised linolenic acid from 20.0 to 39.8 mol% at the expense of linoleic acid, increasing the double bond index from 3.06 to 3.55 [73]. The PE/PC molar ratio rose in parallel, from 1.08 to 1.36, indicating that head-group composition was remodeled alongside acyl desaturation. This shift, however, compensated for only about one-third of the cold-induced loss of membrane fluidity in the tolerant cultivar, and less in the sensitive one; full homeoviscous adaptation was not achieved. Notably, linolenic acid accumulation was not driven by *FAD3* transcription, which rose only transiently, but coincided with a sustained induction of a *SAD* homologue, indicating that desaturation is regulated at several levels.

Lipid-derived signals. Cold-induced lipid remodeling also generates signaling molecules. *B. napus* possesses 32 *PLD* genes, two of which, *BnaPLD*δC7 and *BnaPLD*δ*Ann*, are strongly induced at 4 °C [81]. Phosphatidylcholine declined within hours of cold exposure while phosphatidic acid accumulated transiently, consistent with PLD-mediated PC hydrolysis; PE, by contrast, increased in net output, suggesting that signal generation and structural remodeling proceed in parallel. Functional evidence remains limited, however, manipulation of *BnaPLDα1* alters PA production, but these experiments were performed under non-stress conditions, and a cold-specific role for any *BnaPLD* has yet to be demonstrated.

## 4. Mining Cold-Tolerant Germplasm Resources in Rapeseed

Xian et al. [82] subjected 30 main cultivated varieties (lines) of *Brassica napus* to cold stress at the germination stage (using a fluctuating temperature regime of 10 °C/5 °C to simulate natural low-temperature variation). They used final germination rate, seedling count, primary root length, and cotyledon expansion rate as evaluation indicators and in conjunction with the membership function method, classified cold tolerance levels. Three varieties were identified as strongly cold-tolerant germplasm: Fengyou 823, Dehenyou 999, and Qinyou 7 (Table 1). Zhang et al. [64] based on the climatic characteristics of the main rapeseed production region in the middle reaches of the Yangtze River (Hubei and Hunan provinces), first conducted preliminary trials on 20 extensively cultivated elite varieties. They then selected four accessions with similar growth durations (total growth period 210–220 days) but differing cold tolerance as test materials. Using a field cultivation model (simulating natural overwintering and late spring frost during flowering), they monitored plant cold injury rate, tiller survival rate, and yield traits (siliques per plant, thousand-seed weight) under prolonged low-temperature conditions (overwintering stage: −5~0 °C; flowering stage: 2~8 °C). Combined with physiological indicators such as leaf soluble sugar content and SOD activity, they ultimately identified Huashuang 5 as the variety with the strongest cold tolerance. Jiang et al. [83] established a two-step screening strategy of “field preliminary screening–indoor precision identification”. The first step involved field evaluation of natural chilling injury at the seedling stage for 1145 *Brassica napus* germplasm accessions, classifying them into different chilling injury grades based on leaf chilling injury rate (0–100%), and selecting 42 accessions representing various gradients of chilling injury. In the second step, these 42 accessions underwent indoor low-temperature identification. This included germination-stage experiments at 22 °C (control), 10 °C, and 8 °C (measuring germination rate and radicle length), as well as gradient low-temperature treatments (10 °C, 8 °C, 5 °C) at the 3–4 leaf stage (measuring plant height inhibition rate and root activity). Ultimately, 3 dual-stage comprehensive cold-tolerant germplasms (5223, 7363, 7625) were selected. These accessions combine rapid germination at the germination stage with cold tolerance at the seedling stage, making them suitable for application in early sowing production areas prone to low temperatures. Liu et al. [84] used 49 core germplasm accessions of *Brassica napus* as experimental materials and employed an artificial climate chamber to accurately simulate cold stress at the flowering stage (treatment conditions: −2 °C for 12 h). The core evaluation was based on the degree of plant morphological damage after a 7-day recovery culture following cold stress, including indicators such as petal necrosis rate, stamen fertility, and silique development status. This was combined with the validation of physiological indicators like EL and Malondialdehyde (MDA) content. Ultimately, three strongly freezing-tolerant varieties (JD08, JD10, JD17) were screened out. Wen et al. [85] targeting the low-temperature sensitivity of rapeseed throughout its entire growth cycle, established a “Germination–Flowering” dual-stage cold tolerance screening system. In the first stage, germination was subjected to low-temperature treatment at a constant 8 °C, and germination rate, germination index, and mean germination time were measured, initially screening out 6 early-maturing resources tolerant to low-temperature germination. In the second stage, gradient cold stress during flowering was applied (15 °C/16 h→8 °C/7 h→−2 °C/4 h, simulating the dynamic changes of natural late spring frost), using a grading standard (0–4 levels) to quantify chilling injury symptoms in floral organs (such as stigma desiccation rate and pollen viability). Ultimately, 2 dual-stage cold-tolerant germplasms (D11, D40) were screened. A study initially screened 226 rapeseed varieties/lines under short-term low-temperature and freezing stress. By comparing their cold tolerance performance, two lines were selected: the cold-tolerant line C18 and the cold-sensitive line C6. Specifically, C18 exhibited strong tolerance even after a 3 h gradient freezing treatment ranging from −2 °C to −6 °C, whereas C6 was highly sensitive to low temperature. Subsequently, these two lines were further evaluated under long-term low-temperature conditions (≥2 weeks, 4~8 °C). Observations included phenotypic traits (e.g., leaf growth, degree of chlorosis), assessment of cellular-level damage (e.g., proportion of parenchyma cells showing plasmolysis), and physiological indicators (chlorophyll content, *F_v_*/*F_m_* value, fresh weight, proline content, and electrolyte leakage rate). These analyses confirmed the superior growth and physiological adaptation of C18 under prolonged cold stress, ultimately establishing it as a cold-tolerant germplasm for subsequent molecular mechanism studies [86]. In a study, rapeseed seedlings at the 5–6 leaf stage were used as materials. The experimental procedure was as follows: first, the semi-lethal temperature (LT_50_) was determined by measuring the leaf relative electrolyte leakage at 4 °C, 0 °C, −6 °C, −12 °C, and −20 °C. Based on this, the accession SX9 (with an LT_50_ of −17.14 °C) was preliminarily screened as cold-tolerant. Subsequently, SX9, along with control accessions SX7 and SX8, were subjected to a −10 °C treatment for 1 h (with 0 h as the control). This was followed by physiological index measurements and RNA-seq analysis to elucidate their cold tolerance characteristics and underlying mechanisms. Ultimately, the accession SX9 was identified and selected as the optimal cold-tolerant rapeseed germplasm [87].

In regions such as Canada, Europe, and Australia, the screening of freezing-tolerant *Brassica napus* germplasm mainly follows two routes. In North America and Europe, winter-type materials are used as the research object: greenhouse-grown seedlings are first subjected to sufficient cold acclimation, followed by programmed freezing and recovery, with freezing damage score, survival rate, and chlorophyll fluorescence F_v_/F_m_ as core indicators for classification, combined with genotyping-by-sequencing (GBS) for genome-wide association study (GWAS) mapping. Chao et al. [88] used 399 winter-type *B. napus* accessions from North America/Europe and identified 13 freezing-tolerance loci, with candidate genes involved in lipid metabolism, photosynthesis, and ubiquitination pathways, but no significant association was detected for the CBF gene family. The same team subsequently conducted an independent screen using 222 European winter rapeseed accessions and mapped 11 F_v_/F_m_-associated loci, with the most significant marker on chromosome A01 explaining 12.4% of the phenotypic variation [89]. For spring-type materials, seedling-stage frost screening is emphasized: Fiebelkorn and Rahman [90] established a standardized evaluation protocol in which seedlings are grown at 20 °C for 2 weeks, acclimated at 4 °C for 7 days, exposed to frost at −4 °C, followed by a transitional recovery at 4 °C for 24 h, and scored after 3 days of normal greenhouse cultivation; this protocol is applicable to spring-type *B. napus*-dominant regions such as Australia. In addition, Horvath et al. [91] treated deacclimation resistance as an independent screening target; by comparing freezing tolerance at both the cold-acclimation and deacclimation stages and integrating transcriptome data, they identified five loci associated with post-deacclimation freezing tolerance, providing a genetic basis for breeding resistance to late spring coldness.

Despite heterogeneous conditions, three patterns recur in comparisons between cold-tolerant and sensitive materials during the screening of cold-tolerant germplasm (Table 1). First, in each pair for which physiological data are reported, the tolerant genotype shows lower electrolyte leakage and higher antioxidant capacity and osmotic adjustment under the same stress, with a smaller decline in photosynthesis. Second, the molecular differences are distributed across lipid metabolism, DREB1/CBF-dependent signaling and photosynthetic genes rather than concentrated in a single major locus. Third, tolerance scored at one stage is only partly predictive of performance at another: materials selected at germination or seedling stage do not necessarily retain their advantage at flowering or in the field, which is precisely the information a breeder needs when choosing donors for a target environment. A caveat remains: LT_50_, electrolyte leakage, visual injury scores and germination indices have been used inconsistently across studies, so the magnitude of differences is not directly comparable between reports. Standardized phenotyping at a defined developmental stage, reported together with LT_50_, would make these comparisons directly usable.

## 5. Mining Cold Tolerance Genes in Rapeseed

Genome-Wide Association Study (GWAS), leveraging its advantages of not requiring the construction of artificial populations and directly associating natural variation with phenotypes, has become the mainstream method for analyzing the “gene–phenotype” associations of cold tolerance in *Brassica napus* [92]. Compared to linkage mapping using inbred line populations (requiring the construction of populations like recombinant inbred lines (RILs) or doubled haploids (DHs), which have lower mapping resolution), GWAS can leverage the genetic diversity of natural populations (including core germplasm of different ecotypes and major cultivated varieties) to achieve high-precision mapping of cold-tolerance-related quantitative trait loci (QTL) [93]. In *Brassica napus*, researchers often target key cold tolerance traits such as germination rate under low temperature at the germination stage, the semi-lethal temperature (LT_50_) at the seedling stage, and floral organ survival rate at the flowering stage. The relationship between stress and plant genomic variation can be investigated by mapping related genes through linkage analysis in inbred line populations or by utilizing GWAS [94].

The ICE-CBF-COR cascade is the most conserved and centrally positioned transcriptional pathway regulating cold acclimation in plants. *Brassica napus* is an allotetraploid species with an AACC genome; although this pathway is overall highly conserved, functional divergence exists among multi-copy genes, accompanied by species-specific regulatory features, making it a key research target for dissecting the molecular mechanism of cold tolerance and facilitating cold-tolerant breeding in rapeseed. Affected by genome duplication events, the *CBF* gene family has expanded in *B. napus*. Four tandemly arranged CBF homologs (*BnaCBF1*–*BnaCBF4*) reside on chromosome A03, and more CBF copies are distributed across multiple other chromosomes including C03 [95]. Overexpression of *BnaCBF1* driven by the CaMV 35S promoter in *B. napus* significantly enhances plant freezing tolerance: compared with the wild-type control, electrolyte leakage decreases by 40–60%, and plant survival rate increases by 2–3-fold (*BnaCBF1* was cloned from *B. napus* and functionally validated in both *Arabidopsis* and *B. napus*) [96]. Similarly, overexpression of *BnaCBF3* in *B. napus* upregulates downstream COR genes, thereby enhancing plant cold tolerance [95].

Transcriptomic studies in Arabidopsis have identified more than 100 genes downstream of the CBF, among which the most extensively studied include COR genes such as *COR15a*, *COR47*, and *COR78* [97]. *COR15a* encodes a chloroplast-targeted protein that stabilizes thylakoid membrane structure under freezing conditions by lowering the membrane lipid phase-transition temperature [29,98]. *COR47* (also known as *RD17*) belongs to the dehydrin family and protects cell membranes and proteins from dehydration damage caused by ice crystal formation [90,99]. Homologs of these COR genes have been identified in *B. napus*, including *BnaCOR15a*, *BnaCOR25*, *BnaCOR47*, and *BnaCOR78*; all of these genes are induced by cold stress and participate in cell membrane stabilization and osmotic regulation [95,100].

Phosphorylation provides the first post-translational layer of the cold response. CPK28 is activated within 10 s of cold exposure and phosphorylates NLP7, promoting its nuclear accumulation and COR gene activation [101]; in rice, phosphorylation of OsCNGC9 enhances chilling tolerance [76]. Downstream, OST1 phosphorylates ICE1 at Ser278 and protects it from HOS1-mediated degradation [102], whereas MPK3/MPK6 phosphorylate ICE1 and promote its turnover. The upstream cascades that deliver these phosphorylations, and their integration at ICE1, are considered next [103].

Post-translational modification of the ICE-CBF-COR pathway is regulated by the MAPK signaling cascade. In *Arabidopsis*, MPK3/MPK6 phosphorylate ICE1 protein, mediating its degradation and inhibiting its transcriptional activity, thereby negatively regulating the plant cold response [103,104]. The ubiquitin E3 ligase HOS1 mediates proteasomal degradation of ICE1 under non-stress conditions, exerting negative regulation on this pathway; upon exposure to low temperature, HOS1 activity decreases [104]. Genomic analysis indicates that homologous copies of *MPK3*, *MPK6*, *HOS1*, and *MYB15* exist in *B. napus*, suggesting that the post-translational regulatory module associated with the ICE-CBF pathway is potentially conserved in *Brassica* [94].

The MEKK1-MKK2-MPK4 cascade, in contrast, acts in parallel with MPK3/MPK6 as a positive branch of cold signaling: rather than inhibiting MPK3/MPK6 activity, it phosphorylates ICE1 at distinct sites and thereby counteracts MPK3/MPK6-mediated degradation of ICE1, contributing to cold acclimation [94]. Meanwhile, MPK6 phosphorylates the R2R3-MYB transcription factor MYB15; under ambient temperature, MYB15 directly binds to the *CBF* promoter and represses *CBF* expression. Upon low-temperature activation of MPK6, MYB15 is phosphorylated, its DNA-binding capacity decreases, and transcriptional repression of *CBF* is released. By simultaneously regulating the two substrates ICE1 and MYB15, the MPK pathway coordinately achieves rapid and full activation of CBF under cold stress [24].

WRKY transcription factors also participate in cold tolerance regulation. In *Arabidopsis*, WRKY33 acts as a positive regulator of the cold response, functioning mainly through a CBF-independent pathway by directly activating the expression of downstream *NAC032*, thereby positively regulating plant cold tolerance [105]. WRKY41, in contrast, is a negative regulator that directly binds to the W-box cis-element in the CBF promoter and inhibits its transcription. WRKY41 serves as a substrate of the F-box E3 ligase MAX2; under low-temperature conditions, MAX2 ubiquitinates WRKY41, marking it for cold-induced degradation, thereby alleviating the repression of CBF expression [30].

At present, important progress has been made in exploring and dissecting cold tolerance mechanisms in plants. For example, in rice, the *COLD1* gene was isolated from a cold-tolerance QTL between japonica and indica subspecies via map-based cloning; *COLD1* interacts with the G-protein α subunit *RGA1*; electrophysiological detection confirmed that this complex activates Ca^2+^ channels in response to chilling, leading to cytosolic Ca^2+^ influx; and genetic materials (knockout, overexpression, and natural allelic variants) demonstrated that SNP variations in *COLD1* determine cold tolerance differentiation between japonica and indica subspecies [106,107]. *COLD1* is one of the few membrane cold sensors reported in crops; however, direct biochemical evidence that the protein itself senses low temperature in vitro is still lacking, and it remains difficult to fully distinguish whether the protein directly perceives temperature or perceives low-temperature-induced changes in membrane fluidity. The molecular identity of the downstream calcium channel in this pathway has not yet been fully determined. *Arabidopsis* lacks a *COLD1* homolog, indicating that this sensing module is rice-specific and should not be extrapolated directly to dicot crops. In other crops, such as tomato, it has been found that after 6 h of low-temperature exposure, *SlMYB15* binds to the *SlLOXD* promoter, leading to increased *SlLOXD* expression and JA accumulation while suppressing *SlMYC2* expression. After 24 h of cold stress, sly-miR156e-3p inhibits *SlMYB15* expression, thereby increasing *SlMYC2* expression. Subsequently, SlMYC2 enhances *SlLOXD* promoter activity, promoting the expression of cold-sensitive genes *SlCBF1* and *SlCBF2*. Low temperature greatly affects the yield and quality of crops, and these studies and findings provide a valuable foundation for understanding the molecular mechanisms of crop cold tolerance [108].

In *Brassica rapa*, pharmacological dissection has established a membrane-to-nucleus sequence for cold perception. Chemical rigidification of the plasma membrane with DMSO at 25 °C is sufficient to induce the antifreeze protein gene *BrAFP1*, whereas the fluidizer benzyl alcohol suppresses its cold induction [109]. Microtubule stabilizers block cold activation of *BrAFP1* and *CBF1*–*3*, while microtubule depolymerizers mimic cold-triggered activation [110]. Blocking Ca^2+^ entry with EGTA, La^3+^, Gd^3+^ or ruthenium red strongly reduces transcript levels of *BrAFP1*, *ICE1*, *OST1*, *CNGC* and *CBF*, and exacerbates membrane damage under freezing [111]. Comparable pharmacological evidence in *B. napus* is limited to the *BN115* promoter reporter system [10], and no candidate cold sensor has been functionally characterized in either species.

Although the above studies have revealed multiple cold tolerance mechanisms in crops such as rice and tomato, these findings cannot be directly extrapolated to rapeseed. As an allotetraploid species with an AACC genome, *Brassica napus* has undergone multiple rounds of genome duplication, and its cold tolerance-related genes generally exhibit multi-copy divergence and subfunctionalization. Therefore, it is necessary to dissect the molecular mechanisms of cold tolerance within the genomic context of rapeseed itself. The following section focuses on the key cold-tolerance genes and regulatory pathways that have been identified in *Brassica napus*.

Cold tolerance in *Brassica napus* is governed by a sophisticated, multi-layered regulatory network that operates from transcriptional signal integration at the nucleus to physiological protection at the cellular level. This network can be conceptually organized into four interconnected tiers: upstream transcriptional regulation, midstream antioxidant defense, downstream cellular and membrane protection, and specialized cell-wall metabolic modulation. The following section synthesizes the key cold-tolerance genes identified in *B. napus* within this hierarchical framework, emphasizing their mechanistic interconnections rather than treating them as isolated factors (Table 2).

At the apex of the cold-response regulatory hierarchy lies the ICE-CBF-COR signaling cascade, which serves as the most conserved transcriptional module governing cold acclimation in plants. In *B. napus*, this pathway is reinforced by the atypical bHLH transcription factor *BnaAIF1* (ATBS1 Interacting Factor 1), particularly the *BnaAIF1*-*A03*/*C03* alleles. Under cold stress, *BnaAIF1* is strongly induced, and its encoded protein localizes to the nucleus where it physically interacts with the canonical bHLH transcription factor BnaICE1. This interaction activates the expression of downstream genes in the ICE1-CBF-COR cold-response pathway, thereby enhancing photosystem II stability (elevated F_v_/F_m_ and NPQ_ss_), reducing oxidative damage (decreased H_2_O_2_ and MDA content), and ultimately improving seed germination under cold stress (4 °C) and seedling survival under freezing stress (−4 °C) [96,112]. The identification of *BnaAIF1* as an auxiliary activator of *BnaICE1* underscores the combinatorial nature of transcriptional regulation in *B. napus*, where multiple bHLH family members converge to fine-tune CBF expression.

Counterbalancing this positive regulation, *BnaHOS1* (*High Expression Of Osmotically Responsive Genes 1*), a nuclear pore complex-associated protein, functions as a negative regulator of the cold stress response in *B. napus*. CRISPR/Cas9-mediated editing of *BnaHOS1* in *B. napus* significantly enhances plant cold tolerance, demonstrating that relieving HOS1-mediated repression is an effective strategy for improving cold hardiness and providing a promising candidate gene for molecular breeding [113]. The opposing functions of *BnaAIF1* (positive) and *BnaHOS1* (negative) illustrate that the ICE-CBF-COR axis in *B. napus* is subject to tight bidirectional control, with the net output of CBF expression determined by the balance between activating and degrading forces.

Beyond the core ICE-CBF module, additional transcription factor families contribute to the transcriptional reprogramming underlying cold tolerance. *BnaERF70*, an AP2/ERF family transcription factor, enhances cold tolerance through a multi-dimensional regulatory network that activates key cold-resistance pathways, regulates critical physiological homeostasis, and synergistically integrates multiple signaling networks [49]. Similarly, the *BnaFAR1*/*FHY3* (*Far-Red Impaired Response 1/Far-Red Elongated Hypocotyl 3*) gene family, comprising 21 members in *B. napus*, has been implicated in cold tolerance: qRT-PCR analysis revealed that under cold stress (4 °C), the expression levels of *BnaFAR1*-10, *BnaFAR1*-11, and *BnaFAR1*-21 are significantly upregulated compared with the control (25 °C), with *BnaFAR1*-10 showing the highest induction. All three proteins localize to the nucleus, consistent with their role as transcriptional regulators, suggesting that certain *FAR1*/*FHY3* family members participate in cold tolerance by perceiving low-temperature signals and regulating downstream pathways [47]. The involvement of *FAR1*/*FHY3*, which are classically known as light-signaling regulators, also points to an important cross-talk between light and cold signaling pathways in *B. napus*, as both stimuli converge on the regulation of photosynthetic and stress-responsive gene expression.

A direct consequence of cold stress is the excessive accumulation of reactive oxygen species (ROS), which causes oxidative damage to lipids, proteins, and nucleic acids. To counteract this, plants deploy a sophisticated antioxidant defense system, and *B. napus* harbors expanded gene families encoding the core ROS-scavenging enzymes. The *BnaSOD* (*Superoxide Dismutase*) family comprises 31 gene—including 14 *BnaCSDs* (Cu/Zn-SOD), 11 *BnaFSDs* (Fe-SOD), and 6 *BnaMSDs* (Mn-SOD)—whose core function is to catalyze the dismutation of superoxide radicals (O_2_^−^) into hydrogen peroxide (H_2_O_2_) and molecular oxygen, serving as the first line of defense against oxidative stress. Among these, eight genes—*BnaCSD1*/*3*/*14*, *BnaFSD4*/*5*/*6*, and *BnaMSD2*/*10*—are significantly upregulated under abiotic stresses including low temperature and hormone treatments, enhancing *B. napus* tolerance to cold and other stresses [114]. The SOD-catalyzed conversion of O^2−^ to H_2_O_2_ must be followed by the removal of H_2_O_2_ itself, a task performed primarily by catalases.

The *BnaCAT* (Catalase) family consists of 14 genes encoding catalase enzymes that decompose H_2_O_2_ into water and oxygen, thereby reducing cellular ROS levels and preventing oxidative cell death. Genes such as *BnaCAT1*–*BnaCAT3* and *BnaCAT11*–*BnaCAT13* are significantly upregulated under low-temperature and salt-stress treatments, enhancing *B. napus* tolerance to adverse conditions [115]. The coordinated action of SOD and CAT families thus constitutes a two-step ROS-detoxification cascade: SOD converts the highly reactive superoxide radical to the more stable but still toxic H_2_O_2_, and CAT subsequently eliminates H_2_O_2_ to prevent hydroxyl radical formation via the Fenton reaction.

Connecting the upstream transcriptional regulation to this antioxidant enzymatic machinery is *BnaTR1* (*Tropinone Reductase 1*), a gene identified through associative transcriptomics as a key regulator of freezing-stress adaptability in *B. napus*. *BnaTR1* enhances freezing tolerance through a dual mechanism: on the one hand, it modulates the activities of antioxidant enzymes including SOD, POD, and CAT, thereby directly strengthening the ROS-scavenging capacity described above; on the other hand, it upregulates the expression of stress-responsive transcription factors such as CBF, as well as calcium-dependent protein kinases (CPKs), and may interact with CPK family proteins through ubiquitination modifications [116]. In this way, *BnaTR1* serves as a molecular bridge linking the transcriptional activation of cold-response genes (including the CBF pathway discussed in the previous section) to the enzymatic antioxidant defense system, ensuring that the upregulation of stress-responsive transcription factors is translated into effective ROS clearance at the physiological level. This dual role also highlights that the boundary between transcriptional regulation and antioxidant defense is not absolute, many cold-tolerance genes operate across multiple tiers of the regulatory network.

While transcriptional regulation and antioxidant defense mitigate the upstream signaling and oxidative consequences of cold stress, the ultimate targets of protection are cellular structures, particularly membranes, proteins, and the photosynthetic apparatus. Several gene families in *B. napus* encode proteins that directly stabilize these cellular components under freezing conditions. For example, *BnaA.LEA6.a* (*Subgenome Late Embryogenesis Abundant 6 subgroup a*) encodes a representative member of the LEA protein family, a group of highly hydrophilic, thermally stable proteins characterized by their ability to protect cellular structures under dehydration and freezing stress. *BnaA.LEA6.a* enhances rapeseed tolerance to low temperatures through a multi-dimensional synergistic mechanism: first, it stabilizes cell membrane structures, thereby reducing electrolyte leakage; second, it assists in scavenging reactive oxygen species, mitigating oxidative damage; and third, it functions as a molecular chaperone, protecting proteins and nucleic acids from cold-induced denaturation. Overexpression of *BnaA.LEA6.a* in *B. napus* results in lower relative electrical leakage and higher survival rates under freezing treatment compared with the wild type, confirming its protective role in vivo [117]. The multi-functionality of *BnaA.LEA6.a*—spanning membrane stabilization, ROS scavenging, and molecular chaperoning—illustrates that downstream protective proteins often reinforce the actions of the upstream antioxidant enzymes, creating a layered defense system in which ROS are removed both enzymatically (by SOD/CAT) and non-enzymatically (by LEA proteins).

Another class of cold-protective proteins comprises the cold-regulated (COR) proteins, which are the canonical downstream targets of the CBF transcription factors. In *B. napus*, *BnaCOR25* (*Cold-Regulated Protein 25*) has been functionally characterized as a key player in the abscisic acid (ABA)-dependent cold-response pathway. Functional validation in heterologous systems demonstrated that fission yeast overexpressing *BnaCOR25* exhibited a 1.4- to 2-fold increase in survival rate after freezing at −20 °C for 48 h, while *Arabidopsis thaliana* overexpressing *BnaCOR25* displayed faster root growth than the wild type under 4 °C low temperature, less chlorophyll decline in leaves, and enhanced cold tolerance. These findings indicate that *BnaCOR25* plays a critical role in plant responses to low temperature and osmotic stress, providing genetic resources and theoretical support for crop cold-tolerance breeding [118]. The ABA-dependent regulation of *BnaCOR25* is particularly noteworthy, as it reveals that the COR gene family in *B. napus* is not exclusively controlled by the CBF-dependent (ABA-independent) pathway; instead, both ABA-dependent and ABA-independent signaling routes converge on the activation of COR proteins, ensuring a robust and redundant cold-protective response.

At the level of the photosynthetic apparatus, *BnaLhcb3.4* (*Light-Harvesting Chlorophyll a/b-Binding Protein 3.4*), a novel member of the light-harvesting chlorophyll a/b-binding protein gene family in *B. napus*, regulates cold tolerance primarily through two interconnected mechanisms. First, it maintains the structural stability of chloroplast thylakoid membranes and optimizes the function of photosystem II (PSII), thereby ensuring normal photosynthesis under low temperatures and reducing energy metabolism disruption. Second, it enhances ROS-scavenging mechanisms, thereby decreasing oxidative damage induced by low temperatures. Subcellular localization confirmed that *BnaLhcb3.4* is targeted to the chloroplast, and transgenic *Arabidopsis* overexpressing *BnaLhcb3.4* showed significantly increased freezing tolerance and altered ABA sensitivity, with a set of ABA-responsive genes being differentially expressed, indicating that *BnaLhcb3.4* may contribute to cold tolerance through the ABA signaling pathway [106]. The dual role of *BnaLhcb3.4* in both photosynthetic protection and ROS scavenging reinforces a recurring theme in *B. napus* cold tolerance: the chloroplast is both a primary site of cold-induced damage and a key source of retrograde signals that modulate nuclear gene expression, making chloroplast-localized proteins critical nodes in the cold-response network [119].

**Table 2:** Reported genes associated with cold tolerance in *Brassica napus*, their chromosomal locations and biological functions.

Gene	Chromosome	Function	Evidence in *B. napus*	References
*BnaA.LEA6.a*	A02	Stabilize cell structure and regulate antifreeze pathways	Transgenic OE	[117]
*BnaAIF1*	A03/C03	Bind to *BnaICE1* to activate cold tolerance pathways	Transgenic OE	[112]
*BnaTR1*	A04	Modulates SOD, POD and CAT activities; up-regulates CBF and CPK expression	Heterologous OE (*Arabidopsis*)	[116]
*BnaCOR25*	A05	Mediated via ABA-dependent pathways	Heterologous OE (yeast, *Arabidopsis*)	[118]
*BnaLhcb3.4*	C04	Regulate cold tolerance through ABA-dependent mechanisms in the late stage of freezing stress	Heterologous OE (*Arabidopsis*)	[119]
*BnaHOS1*	A03	RING E3 ligase that ubiquitinates *ICE1* and negatively regulates cold signalling	Transgenic/RNAi	[113]
*Bna-miR397a* /*BnaLAC2*	A06/C06	Regulate lignin remodeling and ROS homeostasis, improve rapeseed low-temperature tolerance	Transgenic OE/STTM/CRISPR	[73]
*BnaERF70*	C07	Activates the CBF/DREB core pathway	Heterologous validation (*Arabidopsis*); expression induced by cold in *B. napus*	[49]
*BnaCBFs* (*BnaCBF5/7/16/17*)	multiple loci (A/C)	AP2/ERF transcription factors are cold-induced and activate COR genes	Expression (cold-induced)	[36,95]
*BnaCORs* (*COR15a/COR47/COR78/COR6.6*)	multiple loci (A/C)	LEA-type and chloroplast-targeted protective proteins that stabilise membranes and proteins during freezing	Expression (cold-induced, CBF-regulated)	[3,29]

In addition to the conserved transcriptional, antioxidant, and cellular protection tiers described above, *B. napus* has evolved a specialized post-transcriptional regulatory module that modulates cell-wall properties to enhance cold tolerance. Through integrated RNA sequencing and small RNA sequencing, the Bna-miR397a-BnaLAC2 module was identified as a key regulator of low-temperature adaptation in *B. napus*. Post-transcriptional cleavage of BnaLAC2 (Laccase 2) mRNA by miR397a (Bna-miR397a) suppresses expression of the encoded laccase. Laccases are multicopper oxidases best known for their role in lignin polymerization; consequently, the miR397a-mediated repression of BnaLAC2 leads to reduced total lignin accumulation and altered lignin monomer composition, specifically, a decreased proportion of G-units, which enhances cell-wall flexibility. A more flexible cell wall is better able to withstand the mechanical stress imposed by extracellular ice crystal formation during freezing, reducing physical damage to cells.

Concurrently, the Bna-miR397a-BnaLAC2 module enhances the ROS-scavenging system (e.g., by increasing the activity of enzymes such as GST and CAT) and activates the CBF/COR cold-response pathway, ultimately improving the cold tolerance of *B. napus*. This module is functionally conserved in Brassicaceae plants (e.g., *Arabidopsis thaliana*), as overexpression of Bna-miR397a in Arabidopsis also improved freezing tolerance, providing critical genetic resources for cold-tolerance breeding in rapeseed [73]. The Bna-miR397a-BnaLAC2 module is particularly instructive because it connects all four tiers of the cold-tolerance network simultaneously: it operates at the post-transcriptional level (miRNA-mediated gene silencing), modulates antioxidant defense (GST/CAT activity), activates the CBF/COR transcriptional pathway, and alters cell-wall biomechanical properties. This multi-level integration exemplifies how a single regulatory module can coordinate diverse physiological outputs to achieve comprehensive cold protection.

## 6. Conclusions

Freezing injury is a significant constraint in rapeseed production. Rapeseed exhibits different responses to low temperatures at various growth stages, specifically manifested as follows: slow germination rate and low germination percentage at the germination stage; leaf yellowing and wilting at the seedling stage; and frost damage to floral organs at the flowering stage. Ultimately, all these effects impact yield. Research on the genetics of cold tolerance is crucial for elucidating the genetic basis of low-temperature responses in rapeseed, mining cold-tolerant germplasm, and breeding cold-tolerant varieties. Currently, the conserved cold tolerance pathway in plants, the ICE-CBF-COR pathway, is being studied [120], certain key genes have been confirmed to play crucial regulatory roles in the cold tolerance process of rapeseed. Simultaneously, researchers have screened some cold-tolerant germplasm resources from rapeseed (Table 1), and have also identified a batch of cold tolerance genes related to cell membrane stability and antioxidant capacity (Table 2). These research findings not only enhance our understanding of the cold tolerance pathways in rapeseed but also provide important material and genetic foundations for the subsequent breeding of cold-tolerant rapeseed varieties.

**Figure 1:**
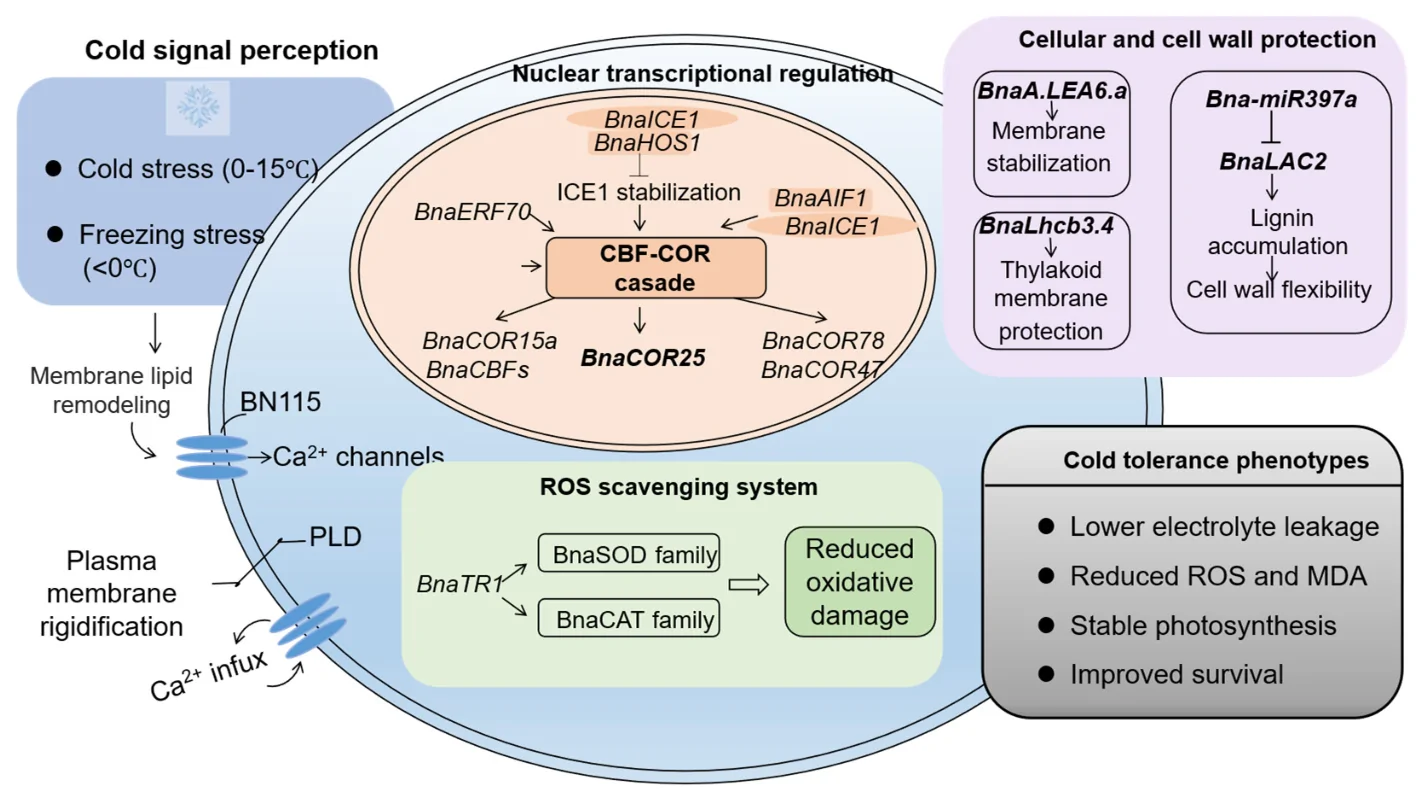
Cold Response in Rapeseed.

Taken together, the cold-tolerance genes identified in *B. napus* do not function in isolation but rather form a convergent, multi-layered regulatory network (Figure 1). At the upstream level, transcription factors including *BnaAIF1*, *BnaHOS1*, *BnERF70*, and *BnaFAR1*/*FHY3* integrate cold signals and modulate the expression of the core ICE-CBF-COR cascade. At the midstream level, the SOD and CAT gene families, coordinated by regulators such as *BnaTR1*, constitute a robust ROS-scavenging system that limits oxidative damage. At the downstream level, LEA proteins (*BnaA.LEA6.a*), COR proteins (*BnCOR25*), and light-harvesting proteins (*BnaLhcb3.4*) directly stabilize membranes, proteins, and the photosynthetic apparatus. And at the specialized level, the Bna-miR397a–BnaLAC2 module modulates cell-wall flexibility while simultaneously reinforcing antioxidant and transcriptional defenses. The extensive cross-talk among these tiers, exemplified by *BnaTR1* linking transcription to antioxidant enzyme activity, *BnaA.LEA6.a* combining membrane stabilization with ROS scavenging, and Bna-miR397a–BnaLAC2 integrating post-transcriptional, metabolic, and transcriptional regulation, ensures that the cold-response network is both robust and flexible. Understanding this integrated regulatory architecture is essential for the rational design of cold-tolerant *B. napus* varieties through molecular breeding [121].

Schematic overview of cold-signaling and cold-tolerance mechanisms in *Brassica napus*. Cold and freezing stress trigger membrane lipid remodeling and plasma-membrane rigidification, which, together with PLD-derived signaling, promote Ca^2+^ influx through Ca^2+^-permeable channels. The Ca^2+^ signal converges on nuclear transcriptional regulation, where ICE1 stabilization, *BnaAIF1* and *BnaERF70* modulate the CBF–COR cascade and drive the expression of CBF and COR genes (*COR15a*, *COR25*, *COR47* and *COR78*). In parallel, *BnaTR1* enhances the SOD and CAT systems and limits oxidative damage, whereas *BnaA.LEA6.a*, *BnaLhcb3.4* and the Bna-miR397a–BnaLAC2 module contribute to membrane stabilization, thylakoid protection, lignin deposition and cell-wall flexibility. These pathways converge on the cold-tolerance phenotypes: lower electrolyte leakage, reduced ROS and MDA accumulation, stable photosynthesis and improved survival. Solid arrows indicate activation; T-shaped connectors indicate repression. Abbreviations: CAT, catalase; EL, electrolyte leakage; MDA, malondialdehyde; PLD, phospholipase D; ROS, reactive oxygen species; SOD, superoxide dismutase. Solid arrows indicate activation or positive regulation, T-shaped connectors indicate repression. Background shading groups functionally related modules: blue, cold perception; orange, nuclear transcriptional regulation; purple, cellular and cell-wall protection; green, ROS scavenging; grey, cold-tolerance phenotypes. 

## 7. Perspectives

Antioxidant responses correlate with cold tolerance only inconsistently. Enzyme activity, protein abundance and transcript levels do not change in parallel [43]; response thresholds differ among enzymes and genotypes [24]; and SOD activity alone provides limited information without corresponding H_2_O_2_-scavenging capacity. Genotype comparisons reveal that tolerant lines combine higher antioxidant capacity with lower MDA content and electrolyte leakage [16,79], although this distinction is most evident at the seedling stage and attenuated during germination. The relative contributions of distinct protective pathways remain poorly quantified: in *Arabidopsis*, CBF-dependent regulation accounts for roughly one-third of acquired freezing tolerance [121], and *COR15a* alone confers approximately 2 °C of freezing tolerance [88]. By contrast, no study in rapeseed has systematically compared the CBF, antioxidant, osmolyte and lipid protective components within a common genetic background.

Currently, in plant cold tolerance research, two main approaches are employed: (1) exploring natural genetic variation between cold-tolerant and sensitive materials; and (2) breeding transgenic materials with novel genes or manipulating the expression levels of existing genes. Current cold tolerance studies primarily integrate phenotypic identification with molecular techniques to decipher the genetic basis of cold tolerance in plants. This is mainly achieved through large-scale screening of cold-tolerant materials to elucidate the genetic basis of cold tolerance genes and their associated regulatory networks.

Despite some achievements in the field of cold tolerance research in *B. napus*, numerous issues require further investigation. On one hand, the genetic mechanisms underlying cold tolerance in *B. napus* are not yet fully elucidated. As an allopolyploid plant, the mechanisms are intricate and complex. Moreover, compared to cold-acclimating plants, the unique molecular pathways by which *B. napus* adapts to low temperatures remain to be fully uncovered, posing challenges for comprehensively deciphering its genetic basis for cold tolerance. There is an urgent need to identify more key cold tolerance genes and analyze their upstream and downstream regulatory networks. On the other hand, research on the impact mechanisms of cold stress on different growth stages of *B. napus* remains relatively shallow. Most studies focus on the germination stage, and existing screening experiments are largely confined to non-lethal low-temperature environments above 0 °C lacking systematic identification and evaluation of rapeseed germplasm’s cold tolerance under extreme cold stress (below 0 °C) during the seedling stage and other periods. In-depth investigation of these mechanisms is of great significance for breeding *B. napus* varieties that can ensure both yield and quality under low-temperature environments.

Due to the significant variation in cold tolerance among different *B. napus* varieties, there is a need to establish unified standards for cold tolerance identification. Future research should focus on three key areas: (1) establishing a system for cold tolerance trait identification and evaluation by developing standardized, high-throughput, and digital technologies for phenotyping and assessment; (2) leveraging multi-omics technologies for a comprehensive analysis of the biological mechanisms underlying cold tolerance to discover key genes; and (3) advancing molecular breeding through the identification of allelic variations in critical cold tolerance genes to create high-quality cold-tolerant germplasm resources, establish efficient molecular breeding technical systems, and apply newly discovered cold tolerance genes in agricultural production to develop new cold-tolerant varieties. By integrating these multidisciplinary technical approaches, we can comprehensively and systematically mine the key genes for cold tolerance in *B. napus* and elucidate its regulatory networks underlying cold tolerance. Conducting research from multiple perspectives enables a more thorough revelation of the impacts of cold stress on various growth stages of *B. napus*, including seed germination, seedling growth, flowering development, and silique, as well as the underlying molecular mechanisms. This, in turn, provides a solid theoretical foundation for precision breeding in *B. napus*. Simultaneously, research should be strengthened on the synergistic effects between cold stress and other environmental factors, such as light duration and intensity, soil moisture content, and soil salinity and alkalinity. For example, in cold, arid, and saline–alkaline regions of northern China, low temperatures often co-occur with soil salinization. Studying the response mechanisms of *B. napus* under the combined stress of both factors will contribute to breeding varieties that can adapt to complex climatic conditions. These varieties would meet the cold tolerance requirements for overwintering rapeseed in the Yangtze River Basin to ensure safe wintering, while also fulfilling the cold tolerance needs for seed germination in northern spring rapeseed regions, ensuring normal seed germination and robust seedling growth under low-temperature environments. Ultimately, this aims to achieve high and stable yields of *B. napus* across different ecological zones and under complex climatic conditions.

## Figures and Tables

**Table 1:** Representative cold-tolerant *Brassica napus* germplasm resources identified in China, together with evaluation conditions and cold-resistance indices.

Stage	Condition	Germplasm	Evaluation Criteria	References
Germination	Diurnal temperature variation 10 °C/5 °C	Fengyou 823, Deheyou 999, Qinyou 7	Final germination rate	[82]
	10 °C/8 °C	5223, 7363, 7625	Germination rate	[83]
	8 °C	D11, D40	Germination rate	[85]
	−10 °C	SX9	LT_50_	[87]
Seedling	Short-term: −2~6 °C freezing for 3 h; long-term: 4~8 °C for ≥2 weeks	C18	Leaf phenotype, chlorosis degree	[86]
	10 °C, 8 °C, 5 °C	5223, 7363, 7625	Leaf chilling injury	[83]
	2~8 °C	Huashuang 5	Cold injury rate	[77]
Flowering	−2 °C 12 h	JD08, JD10, JD17	Morphology after recovery	[84]
	15 °C/16 h→8 °C/7 h→−2 °C/4 h	D11, D40	Floral organ injury score	[85]

## Data Availability

No new data were created or analyzed in this study. Data sharing is not applicable to this article.
